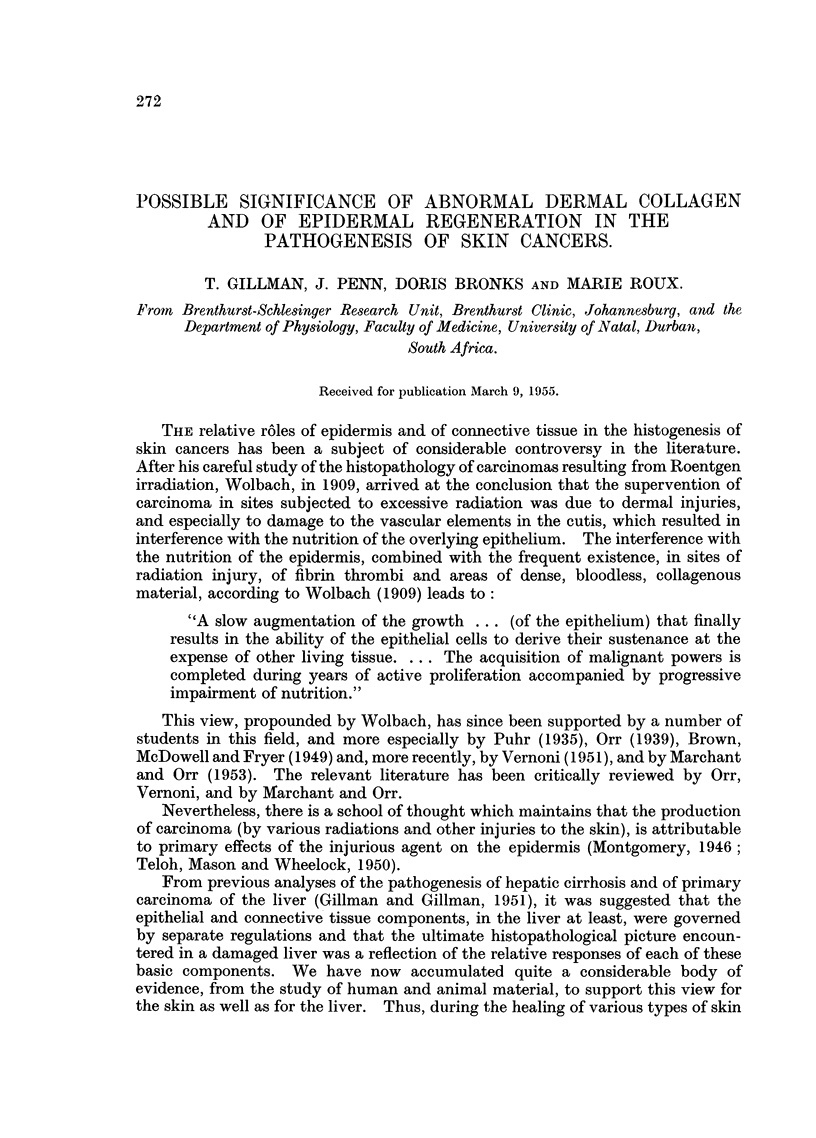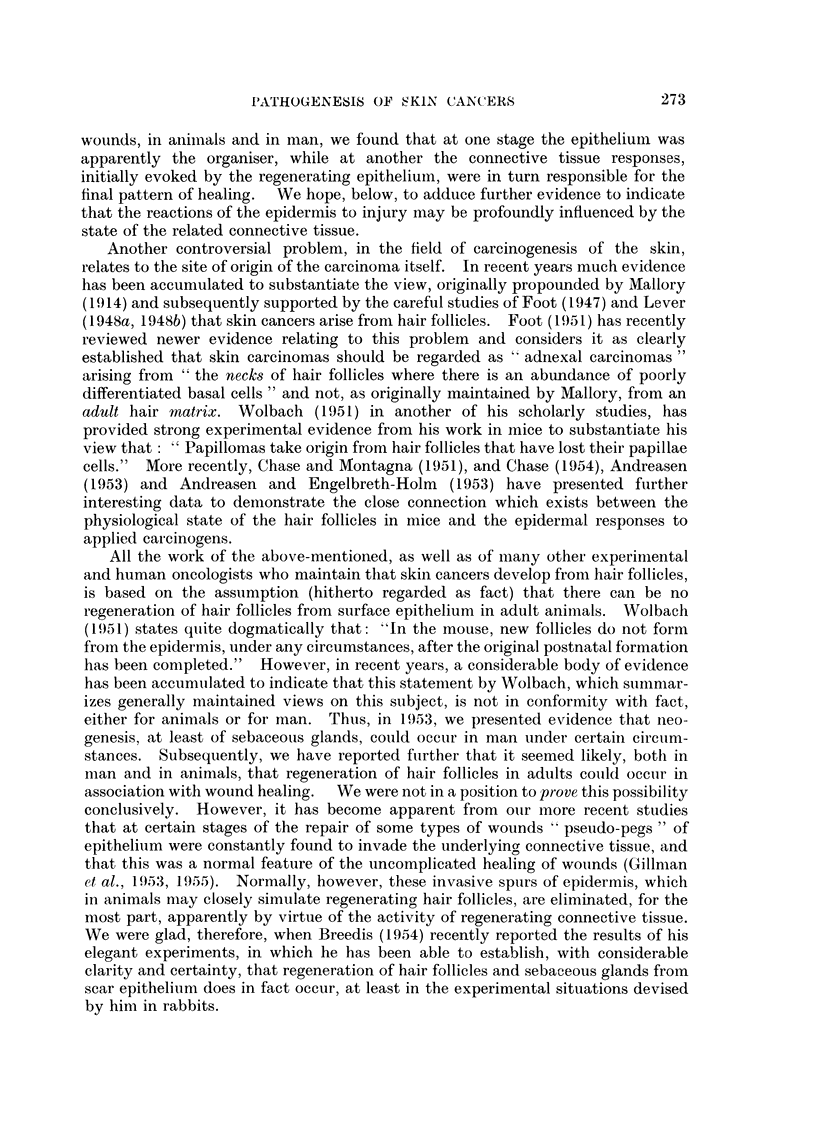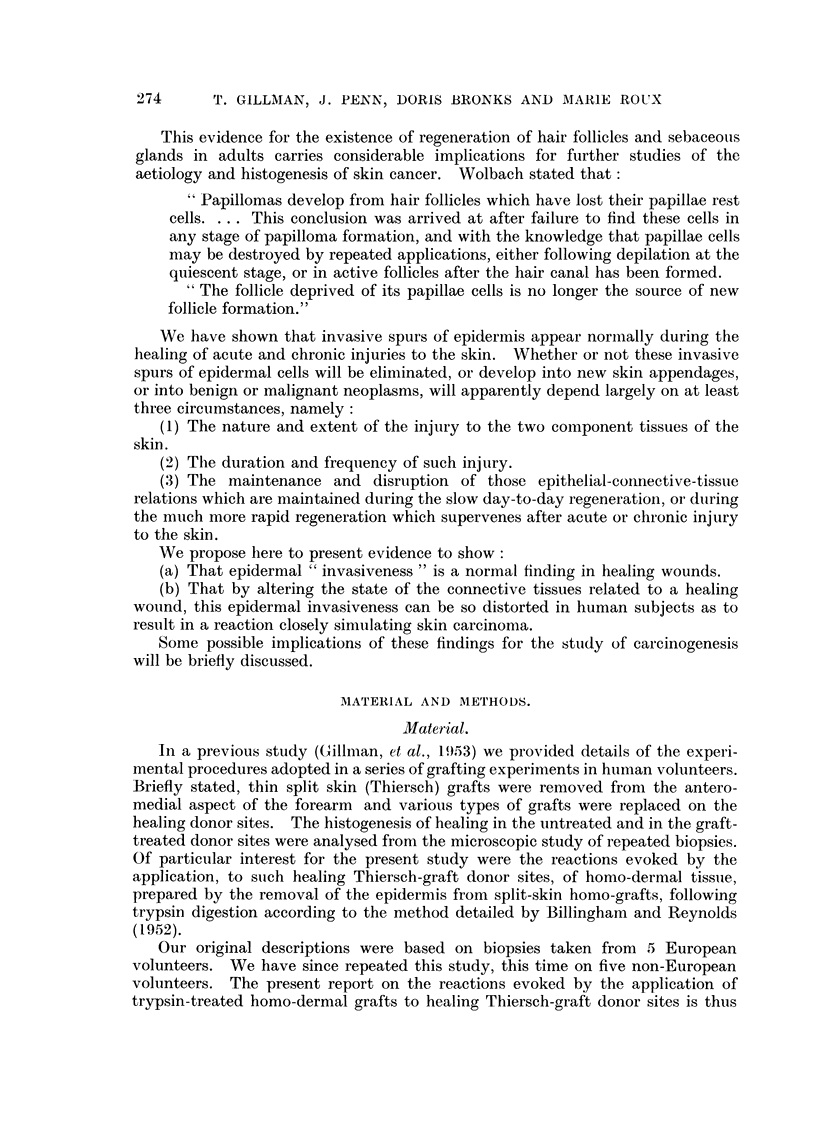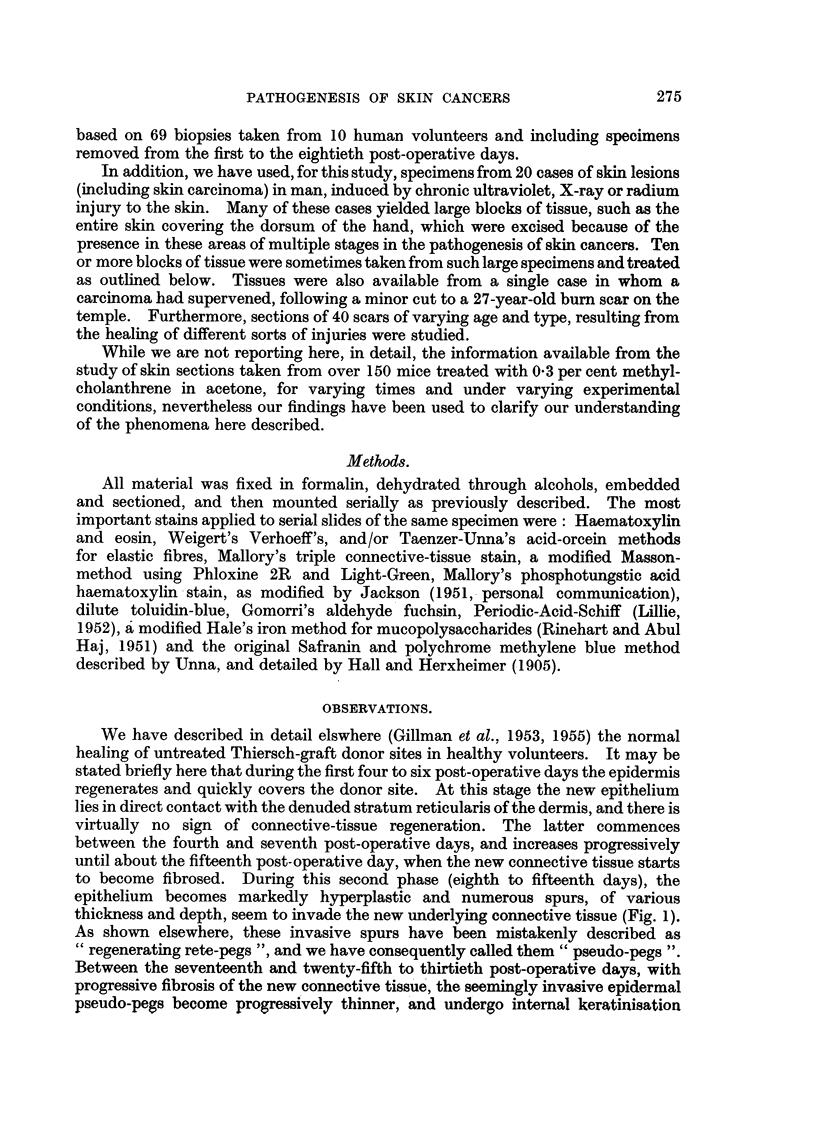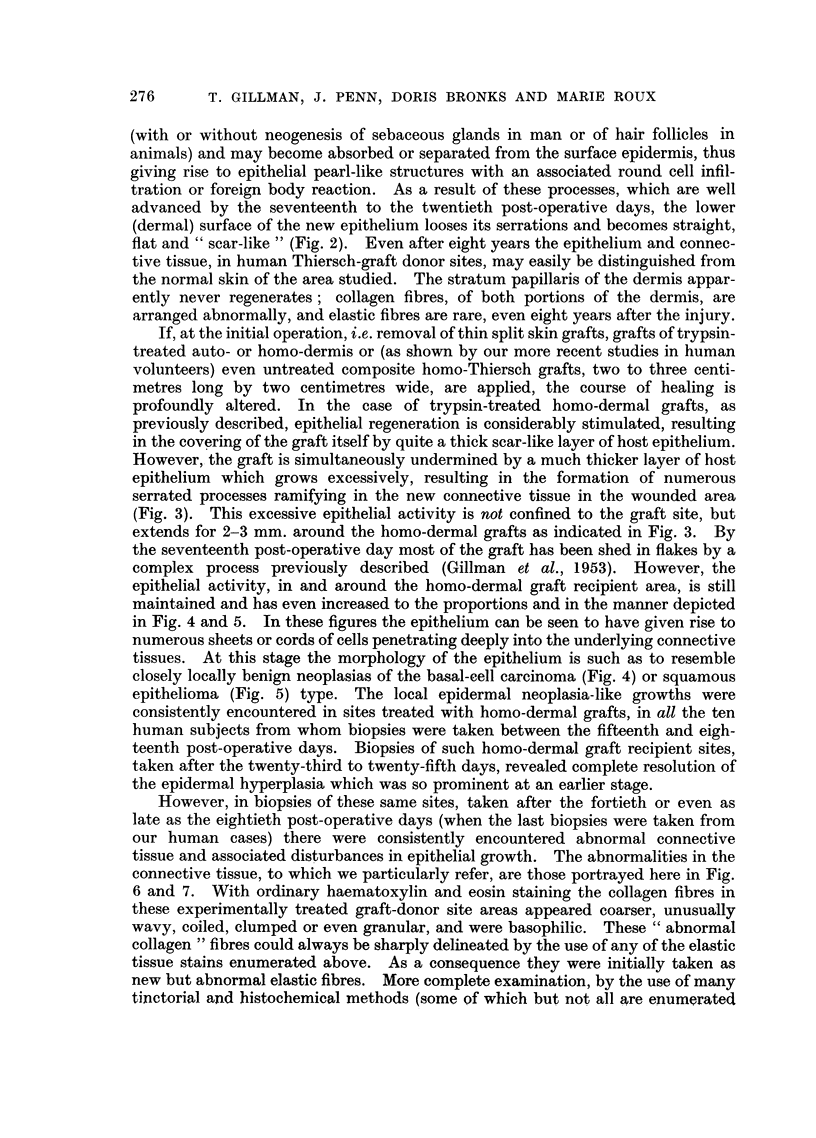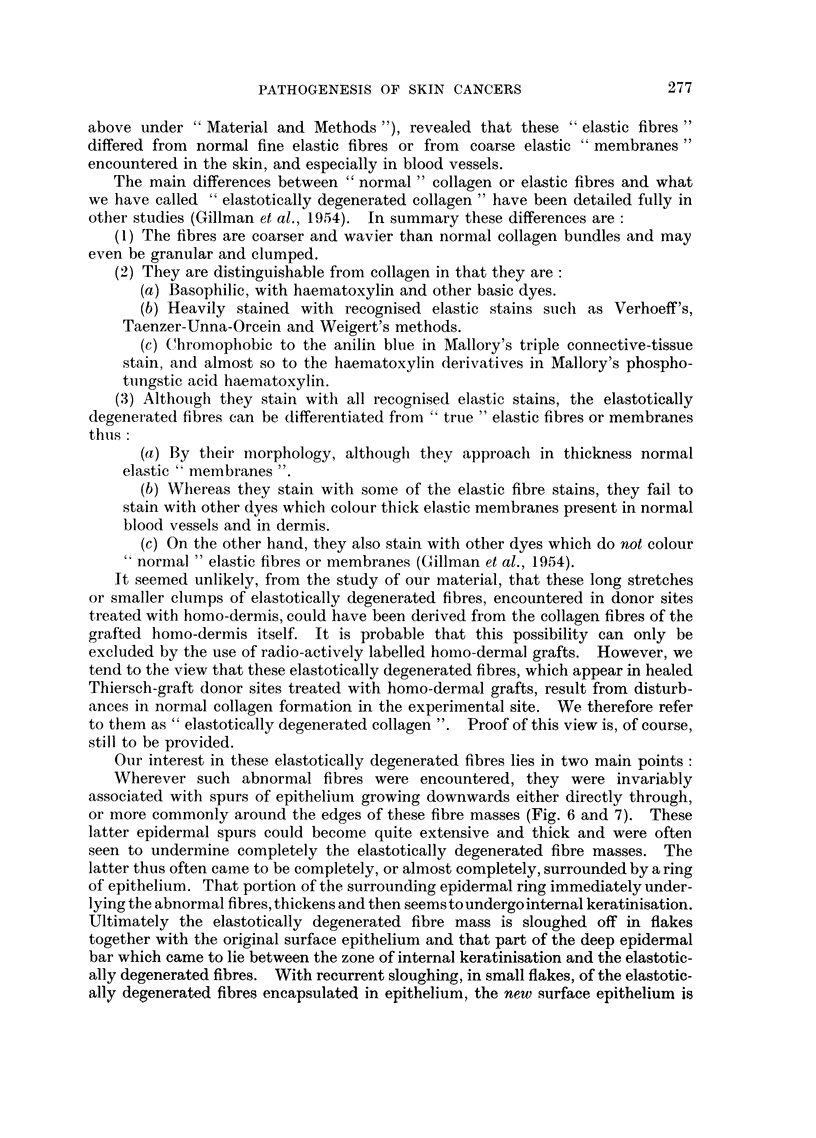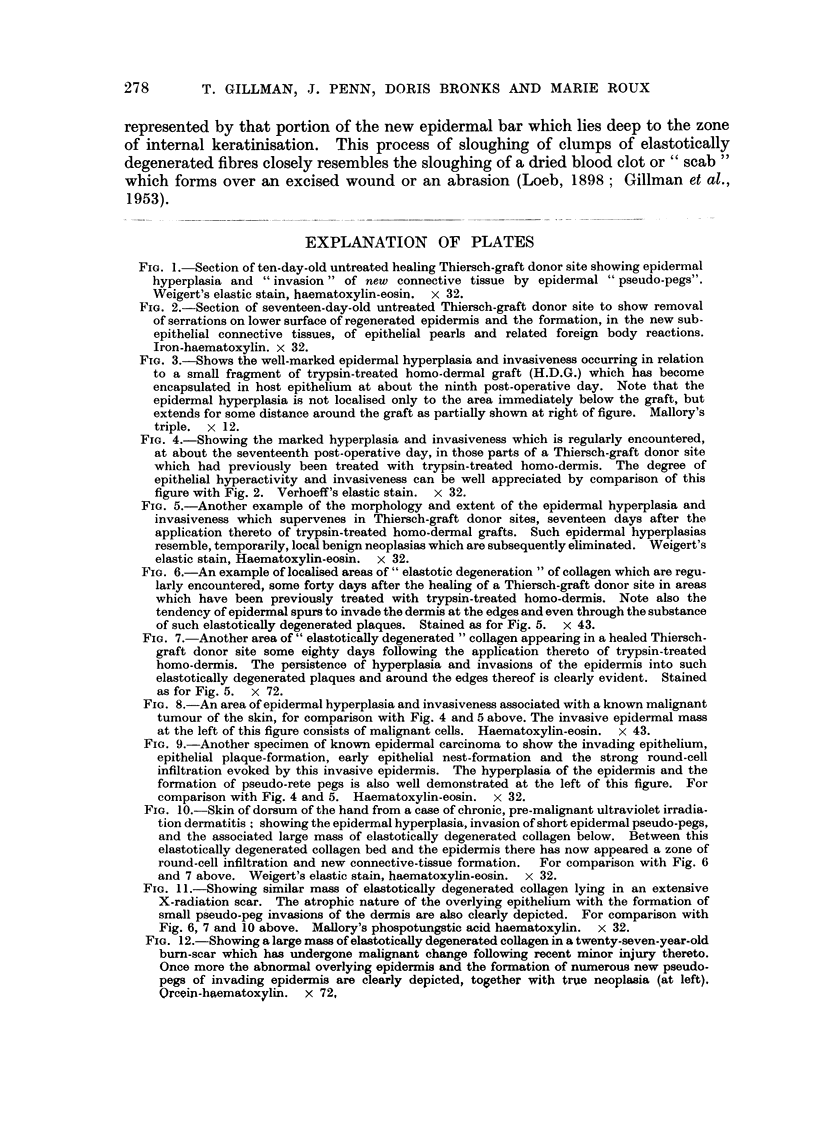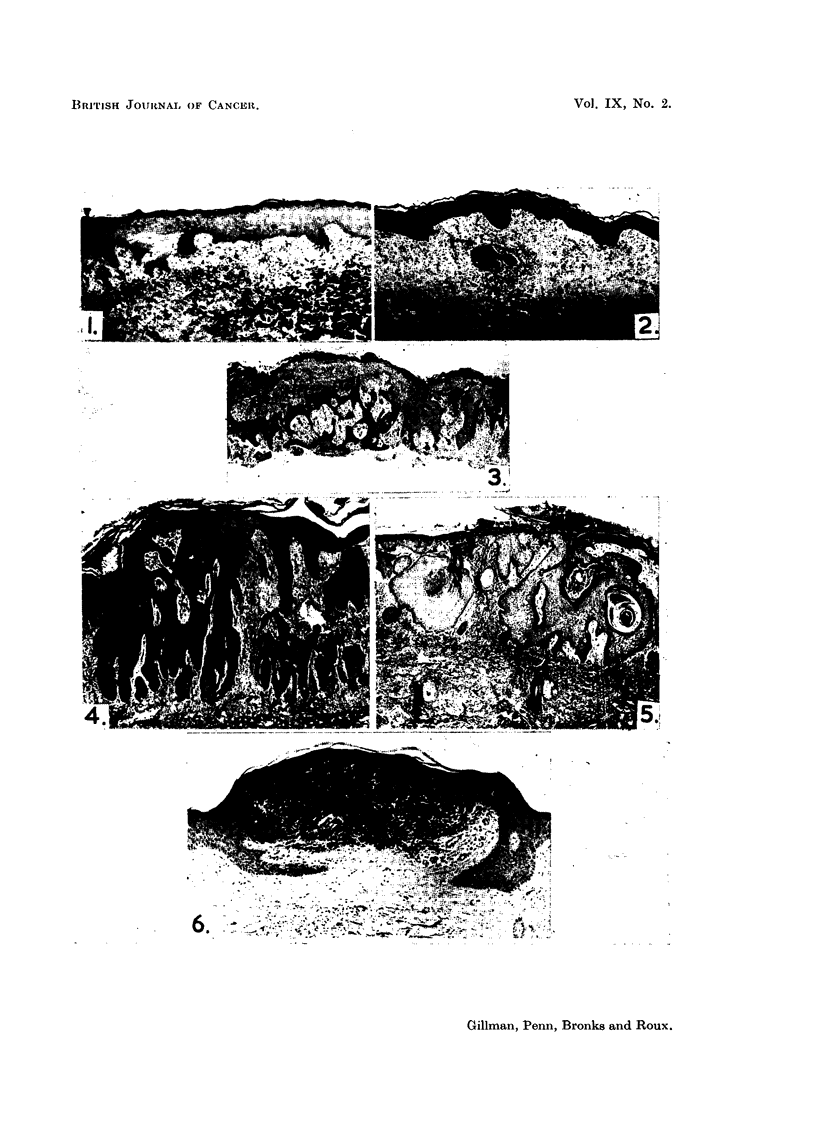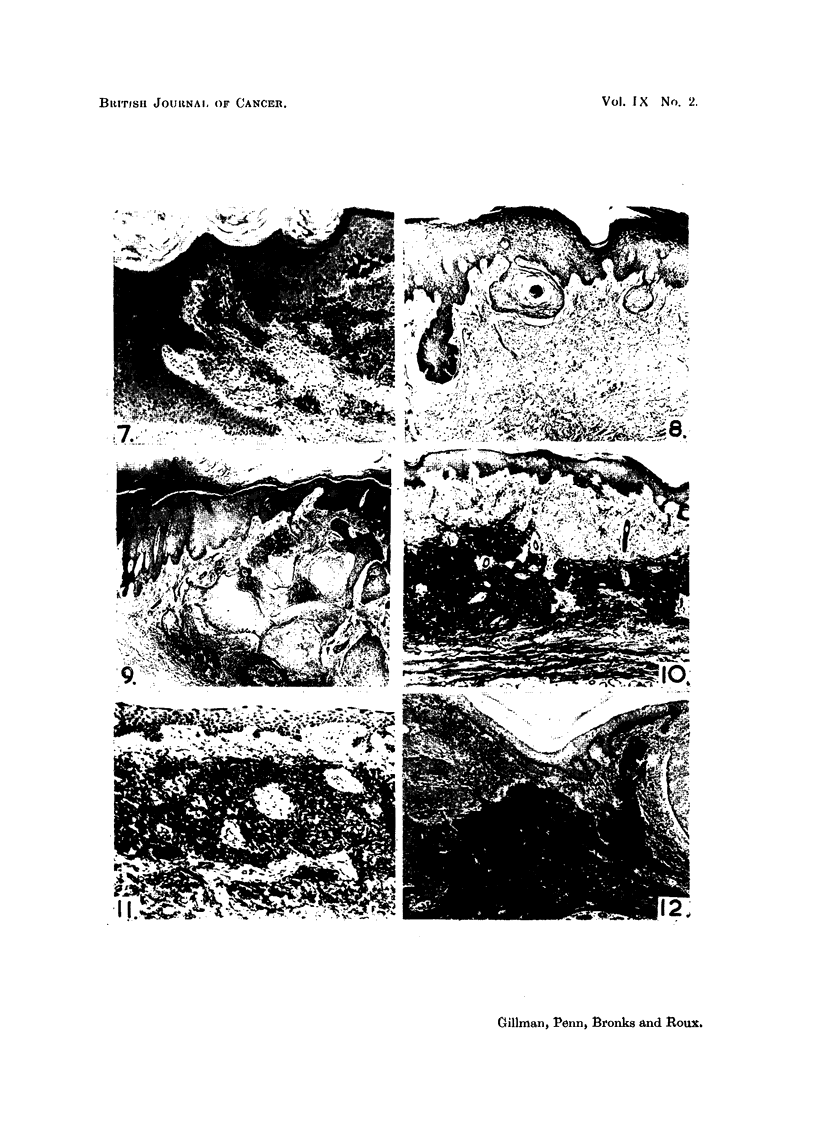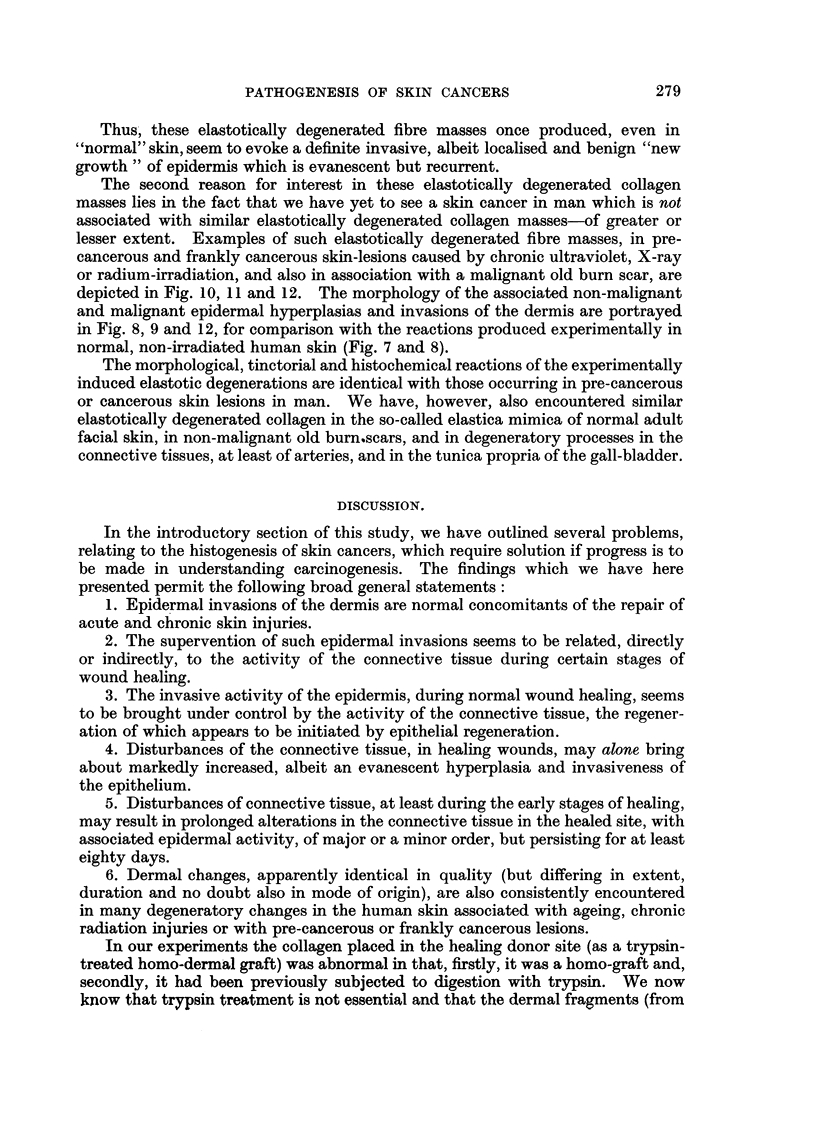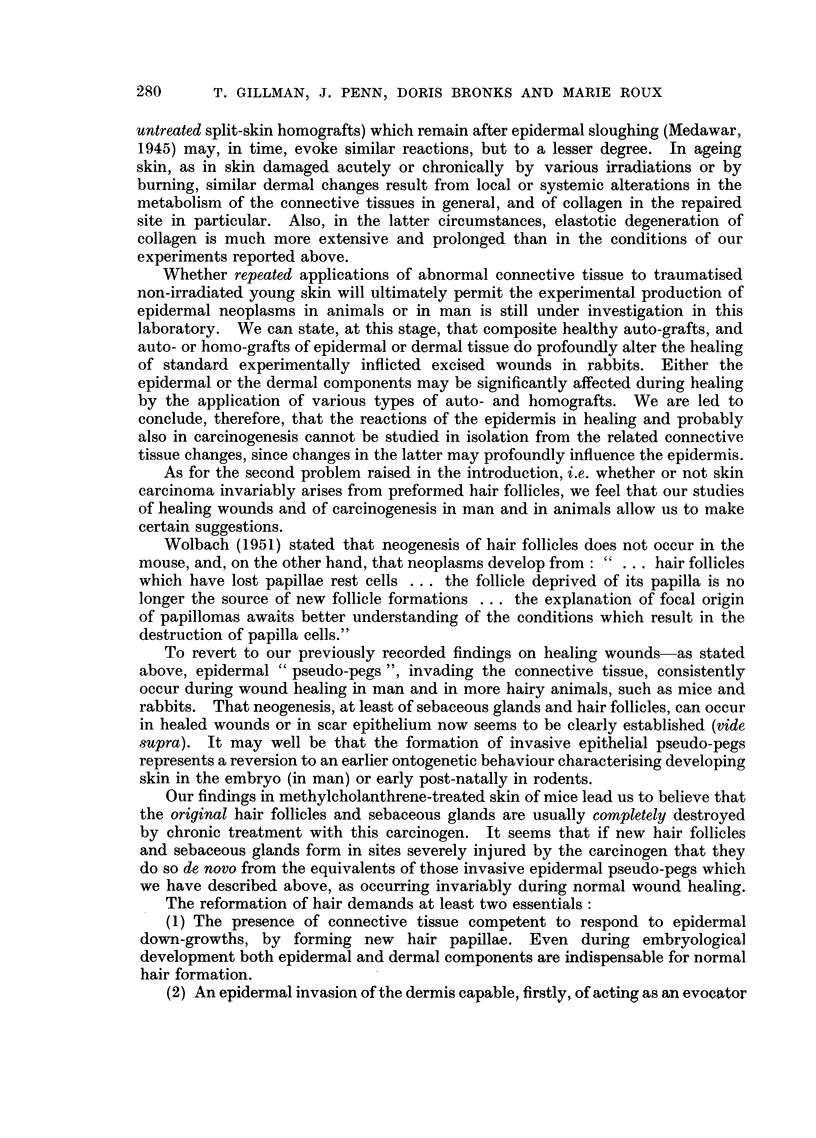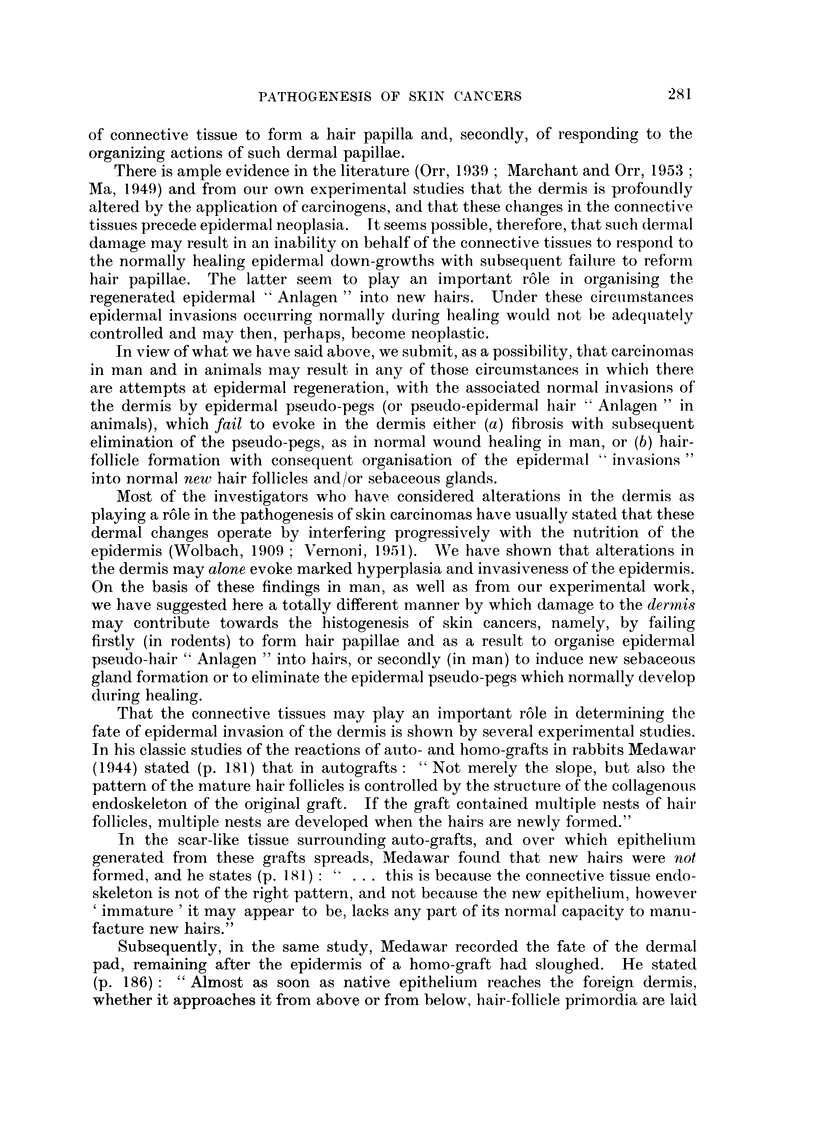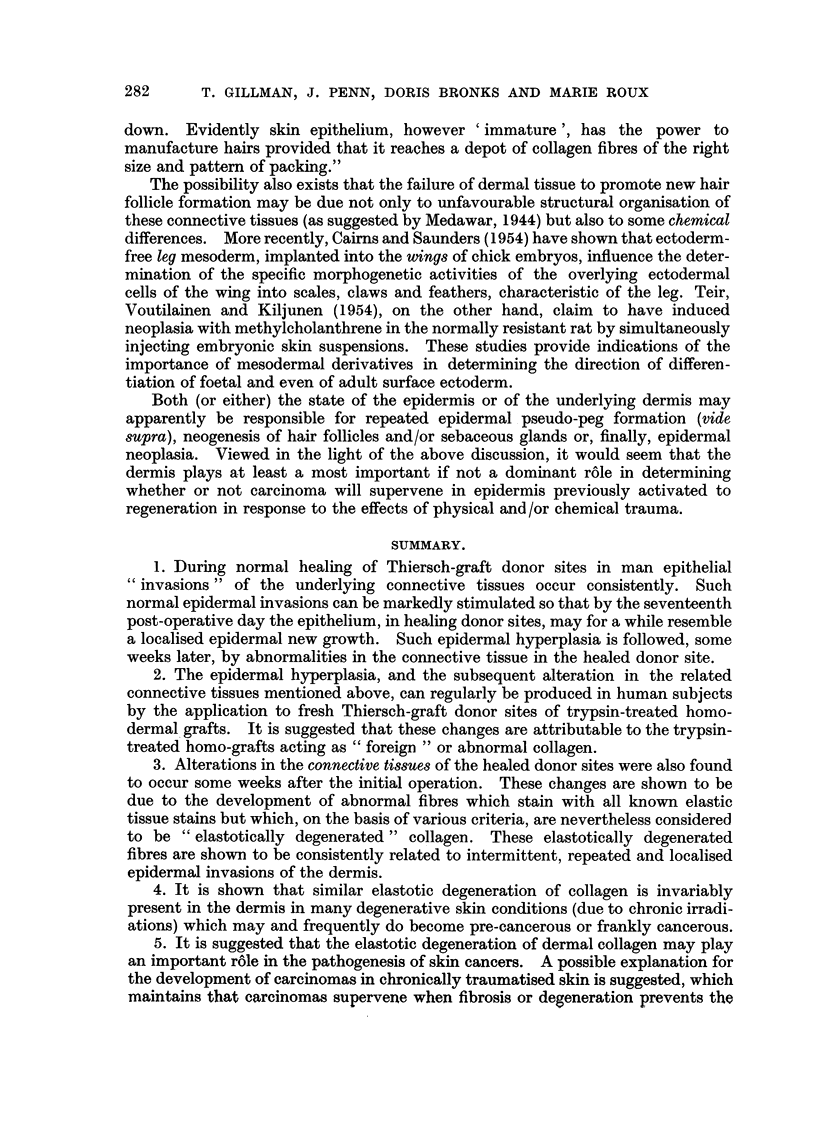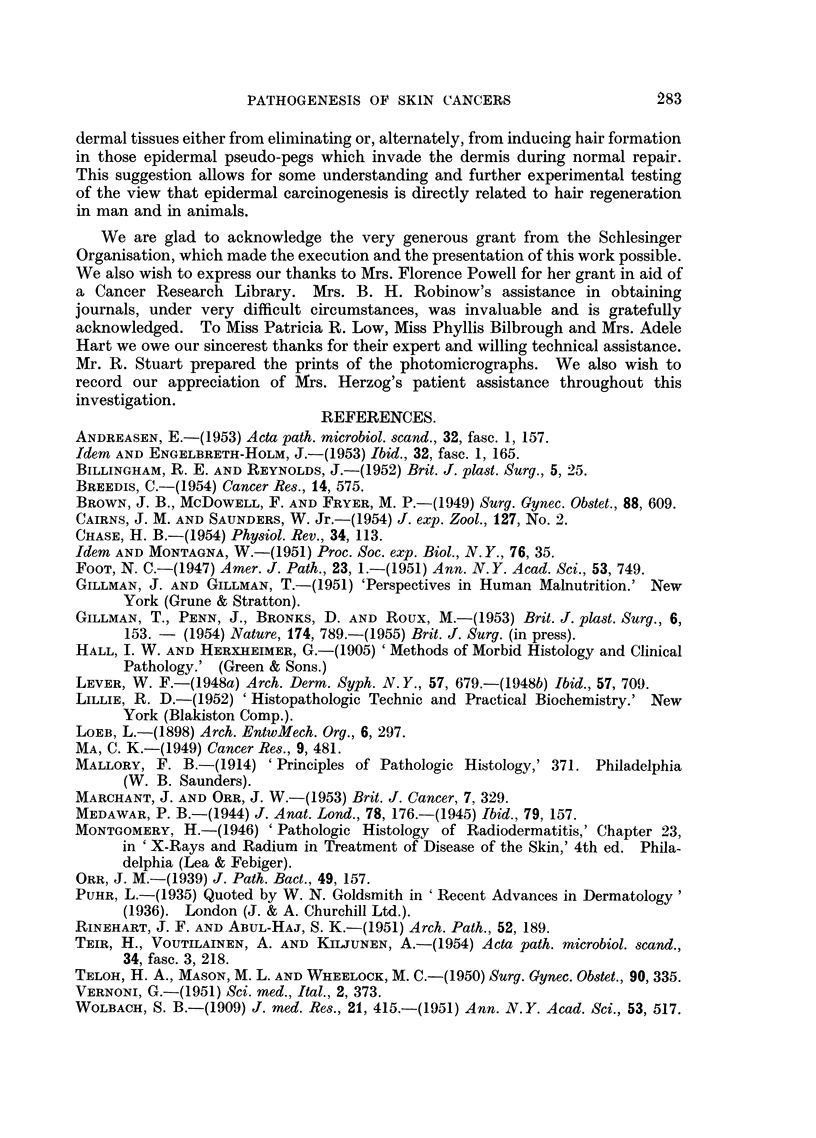# Possible Significance of Abnormal Dermal Collagen and of Epidermal Regeneration in the Pathogenesis of Skin Cancers

**DOI:** 10.1038/bjc.1955.24

**Published:** 1955-06

**Authors:** T. Gillman, J. Penn, Doris Bronks, Marie Roux

## Abstract

**Images:**


### POSSIBLE SIGNIFICANCE OF ABNORMAL DERMAL COLLAGEN AND OF EPIDERMAL REGENERATION IN THE PATHOGENESIS OF SKIN CANCERS.

T. GILLMAN, J. PENN, DORIS BRONKS AND MARIE ROUX.

*From Brenthurst-Schlesinger Research Unit, Brenthurst Clinic, Johannesburg, and the Department of Physiology, Faculty of Medicine, University of Natal, Durban, South Africa.*

Received for publication March 9, 1955.

THE relative rôles of epidermis and of connective tissue in the histogenesis of skin cancers has been a subject of considerable controversy in the literature. After his careful study of the histopathology of carcinomas resulting from Roentgen irradiation, Wolbach, in 1909, arrived at the conclusion that the supervention of carcinoma in sites subjected to excessive radiation was due to dermal injuries, and especially to damage to the vascular elements in the cutis, which resulted in interference with the nutrition of the overlying epithelium. The interference with the nutrition of the epidermis, combined with the frequent existence, in sites of radiation injury, of fibrin thrombi and areas of dense, bloodless, collagenous material, according to Wolbach (1909) leads to :

> "A slow augmentation of the growth ... (of the epithelium) that finally results in the ability of the epithelial cells to derive their sustenance at the expense of other living tissue. ... The acquisition of malignant powers is completed during years of active proliferation accompanied by progressive impairment of nutrition."

This view, propounded by Wolbach, has since been supported by a number of students in this field, and more especially by Puhr (1935), Orr (1939), Brown, McDowell and Fryer (1949) and, more recently, by Vernoni (1951), and by Marchant and Orr (1953). The relevant literature has been critically reviewed by Orr, Vernoni, and by Marchant and Orr.

Nevertheless, there is a school of thought which maintains that the production of carcinoma (by various radiations and other injuries to the skin), is attributable to primary effects of the injurious agent on the epidermis (Montgomery, 1946 ; Teloh, Mason and Wheelock, 1950).

From previous analyses of the pathogenesis of hepatic cirrhosis and of primary carcinoma of the liver (Gillman and Gillman, 1951), it was suggested that the epithelial and connective tissue components, in the liver at least, were governed by separate regulations and that the ultimate histopathological picture encountered in a damaged liver was a reflection of the relative responses of each of these basic components. We have now accumulated quite a considerable body of evidence, from the study of human and animal material, to support this view for the skin as well as for the liver. Thus, during the healing of various types of skin wounds, in animals and in man, we found that at one stage the epithelium was apparently the organiser, while at another the connective tissue responses, initially evoked by the regenerating epithelium, were in turn responsible for the final pattern of healing. We hope, below, to adduce further evidence to indicate that the reactions of the epidermis to injury may be profoundly influenced by the state of the related connective tissue.

Another controversial problem, in the field of carcinogenesis of the skin, relates to the site of origin of the carcinoma itself. In recent years much evidence has been accumulated to substantiate the view, originally propounded by Mallory (1914) and subsequently supported by the careful studies of Foot (1947) and Lever (1948*a*, 1948*b*) that skin cancers arise from hair follicles. Foot (1951) has recently reviewed newer evidence relating to this problem and considers it as clearly established that skin carcinomas should be regarded as " adnexal carcinomas " arising from " the *necks* of hair follicles where there is an abundance of poorly differentiated basal cells " and not, as originally maintained by Mallory, from an *adult* hair *matrix*. Wolbach (1951) in another of his scholarly studies, has provided strong experimental evidence from his work in mice to substantiate his view that : " Papillomas take origin from hair follicles that have lost their papillae cells." More recently, Chase and Montagna (1951), and Chase (1954), Andreasen (1953) and Andreasen and Engelbreth-Holm (1953) have presented further interesting data to demonstrate the close connection which exists between the physiological state of the hair follicles in mice and the epidermal responses to applied carcinogens.

All the work of the above-mentioned, as well as of many other experimental and human oncologists who maintain that skin cancers develop from hair follicles, is based on the assumption (hitherto regarded as fact) that there can be no regeneration of hair follicles from surface epithelium in adult animals. Wolbach (1951) states quite dogmatically that: "In the mouse, new follicles do not form from the epidermis, under any circumstances, after the original postnatal formation has been completed." However, in recent years, a considerable body of evidence has been accumulated to indicate that this statement by Wolbach, which summarizes generally maintained views on this subject, is not in conformity with fact, either for animals or for man. Thus, in 1953, we presented evidence that neogenesis, at least of sebaceous glands, could occur in man under certain circumstances. Subsequently, we have reported further that it seemed likely, both in man and in animals, that regeneration of hair follicles in adults could occur in association with wound healing. We were not in a position to *prove* this possibility conclusively. However, it has become apparent from our more recent studies that at certain stages of the repair of some types of wounds " pseudo-pegs " of epithelium were constantly found to invade the underlying connective tissue, and that this was a normal feature of the uncomplicated healing of wounds (Gillman *et al.*, 1953, 1955). Normally, however, these invasive spurs of epidermis, which in animals may closely simulate regenerating hair follicles, are eliminated, for the most part, apparently by virtue of the activity of regenerating connective tissue. We were glad, therefore, when Breedis (1954) recently reported the results of his elegant experiments, in which he has been able to establish, with considerable clarity and certainty, that regeneration of hair follicles and sebaceous glands from scar epithelium does in fact occur, at least in the experimental situations devised by him in rabbits.

This evidence for the existence of regeneration of hair follicles and sebaceous glands in adults carries considerable implications for further studies of the aetiology and histogenesis of skin cancer. Wolbach stated that :

> " Papillomas develop from hair follicles which have lost their papillae rest cells. . . . This conclusion was arrived at after failure to find these cells in any stage of papilloma formation, and with the knowledge that papillae cells may be destroyed by repeated applications, either following depilation at the quiescent stage, or in active follicles after the hair canal has been formed.
>
> " The follicle deprived of its papillae cells is no longer the source of new follicle formation."

We have shown that invasive spurs of epidermis appear normally during the healing of acute and chronic injuries to the skin. Whether or not these invasive spurs of epidermal cells will be eliminated, or develop into new skin appendages, or into benign or malignant neoplasms, will apparently depend largely on at least three circumstances, namely :

(1) The nature and extent of the injury to the two component tissues of the skin.

(2) The duration and frequency of such injury.

(3) The maintenance and disruption of those epithelial-connective-tissue relations which are maintained during the slow day-to-day regeneration, or during the much more rapid regeneration which supervenes after acute or chronic injury to the skin.

We propose here to present evidence to show :

(a) That epidermal " invasiveness " is a normal finding in healing wounds.

(b) That by altering the state of the connective tissues related to a healing wound, this epidermal invasiveness can be so distorted in human subjects as to result in a reaction closely simulating skin carcinoma.

Some possible implications of these findings for the study of carcinogenesis will be briefly discussed.

### MATERIAL AND METHODS.

### *Material.*

In a previous study (Gillman, *et al.*, 1953) we provided details of the experimental procedures adopted in a series of grafting experiments in human volunteers. Briefly stated, thin split skin (Thiersch) grafts were removed from the antero-medial aspect of the forearm and various types of grafts were replaced on the healing donor sites. The histogenesis of healing in the untreated and in the graft-treated donor sites were analysed from the microscopic study of repeated biopsies. Of particular interest for the present study were the reactions evoked by the application, to such healing Thiersch-graft donor sites, of homo-dermal tissue, prepared by the removal of the epidermis from split-skin homo-grafts, following trypsin digestion according to the method detailed by Billingham and Reynolds (1952).

Our original descriptions were based on biopsies taken from 5 European volunteers. We have since repeated this study, this time on five non-European volunteers. The present report on the reactions evoked by the application of trypsin-treated homo-dermal grafts to healing Thiersch-graft donor sites is thus based on 69 biopsies taken from 10 human volunteers and including specimens removed from the first to the eightieth post-operative days.

In addition, we have used, for this study, specimens from 20 cases of skin lesions (including skin carcinoma) in man, induced by chronic ultraviolet, X-ray or radium injury to the skin. Many of these cases yielded large blocks of tissue, such as the entire skin covering the dorsum of the hand, which were excised because of the presence in these areas of multiple stages in the pathogenesis of skin cancers. Ten or more blocks of tissue were sometimes taken from such large specimens and treated as outlined below. Tissues were also available from a single case in whom a carcinoma had supervened, following a minor cut to a 27-year-old burn scar on the temple. Furthermore, sections of 40 scars of varying age and type, resulting from the healing of different sorts of injuries were studied.

While we are not reporting here, in detail, the information available from the study of skin sections taken from over 150 mice treated with 0·3 per cent methylcholanthrene in acetone, for varying times and under varying experimental conditions, nevertheless our findings have been used to clarify our understanding of the phenomena here described.

### *Methods.*

All material was fixed in formalin, dehydrated through alcohols, embedded and sectioned, and then mounted serially as previously described. The most important stains applied to serial slides of the same specimen were : Haematoxylin and eosin, Weigert's Verhoeff's, and/or Taenzer-Unna's acid-orcein methods for elastic fibres, Mallory's triple connective-tissue stain, a modified Masson-method using Phloxine 2R and Light-Green, Mallory's phosphotungstic acid haematoxylin stain, as modified by Jackson (1951, personal communication), dilute toluidin-blue, Gomorri's aldehyde fuchsin, Periodic-Acid-Schiff (Lillie, 1952), a modified Hale's iron method for mucopolysaccharides (Rinehart and Abul Haj, 1951) and the original Safranin and polychrome methylene blue method described by Unna, and detailed by Hall and Herxheimer (1905).

### OBSERVATIONS.

We have described in detail elswhere (Gillman *et al.*, 1953, 1955) the normal healing of untreated Thiersch-graft donor sites in healthy volunteers. It may be stated briefly here that during the first four to six post-operative days the epidermis regenerates and quickly covers the donor site. At this stage the new epithelium lies in direct contact with the denuded stratum reticularis of the dermis, and there is virtually no sign of connective-tissue regeneration. The latter commences between the fourth and seventh post-operative days, and increases progressively until about the fifteenth post-operative day, when the new connective tissue starts to become fibrosed. During this second phase (eighth to fifteenth days), the epithelium becomes markedly hyperplastic and numerous spurs, of various thickness and depth, seem to invade the new underlying connective tissue (Fig. 1). As shown elsewhere, these invasive spurs have been mistakenly described as " regenerating rete-pegs ", and we have consequently called them " pseudo-pegs ". Between the seventeenth and twenty-fifth to thirtieth post-operative days, with progressive fibrosis of the new connective tissue, the seemingly invasive epidermal pseudo-pegs become progressively thinner, and undergo internal keratinisation

(with or without neogenesis of sebaceous glands in man or of hair follicles in animals) and may become absorbed or separated from the surface epidermis, thus giving rise to epithelial pearl-like structures with an associated round cell infiltration or foreign body reaction. As a result of these processes, which are well advanced by the seventeenth to the twentieth post-operative days, the lower (dermal) surface of the new epithelium looses its serrations and becomes straight, flat and " scar-like " (Fig. 2). Even after eight years the epithelium and connective tissue, in human Thiersch-graft donor sites, may easily be distinguished from the normal skin of the area studied. The stratum papillaris of the dermis apparently never regenerates ; collagen fibres, of both portions of the dermis, are arranged abnormally, and elastic fibres are rare, even eight years after the injury.

If, at the initial operation, *i.e.* removal of thin split skin grafts, grafts of trypsin-treated auto- or homo-dermis or (as shown by our more recent studies in human volunteers) even untreated composite homo-Thiersch grafts, two to three centimetres long by two centimetres wide, are applied, the course of healing is profoundly altered. In the case of trypsin-treated homo-dermal grafts, as previously described, epithelial regeneration is considerably stimulated, resulting in the covering of the graft itself by quite a thick scar-like layer of host epithelium. However, the graft is simultaneously undermined by a much thicker layer of host epithelium which grows excessively, resulting in the formation of numerous serrated processes ramifying in the new connective tissue in the wounded area (Fig. 3). This excessive epithelial activity is *not* confined to the graft site, but extends for 2–3 mm. around the homo-dermal grafts as indicated in Fig. 3. By the seventeenth post-operative day most of the graft has been shed in flakes by a complex process previously described (Gillman *et al.*, 1953). However, the epithelial activity, in and around the homo-dermal graft recipient area, is still maintained and has even increased to the proportions and in the manner depicted in Fig. 4 and 5. In these figures the epithelium can be seen to have given rise to numerous sheets or cords of cells penetrating deeply into the underlying connective tissues. At this stage the morphology of the epithelium is such as to resemble closely locally benign neoplasias of the basal-cell carcinoma (Fig. 4) or squamous epithelioma (Fig. 5) type. The local epidermal neoplasia-like growths were consistently encountered in sites treated with homo-dermal grafts, in *all* the ten human subjects from whom biopsies were taken between the fifteenth and eighteenth post-operative days. Biopsies of such homo-dermal graft recipient sites, taken after the twenty-third to twenty-fifth days, revealed complete resolution of the epidermal hyperplasia which was so prominent at an earlier stage.

However, in biopsies of these same sites, taken after the fortieth or even as late as the eightieth post-operative days (when the last biopsies were taken from our human cases) there were consistently encountered abnormal connective tissue and associated disturbances in epithelial growth. The abnormalities in the connective tissue, to which we particularly refer, are those portrayed here in Fig. 6 and 7. With ordinary haematoxylin and eosin staining the collagen fibres in these experimentally treated graft-donor site areas appeared coarser, unusually wavy, coiled, clumped or even granular, and were basophilic. These " abnormal collagen " fibres could always be sharply delineated by the use of any of the elastic tissue stains enumerated above. As a consequence they were initially taken as new but abnormal elastic fibres. More complete examination, by the use of many tinctorial and histochemical methods (some of which but not all are enumerated above under " Material and Methods "), revealed that these " elastic fibres " differed from normal fine elastic fibres or from coarse elastic " membranes " encountered in the skin, and especially in blood vessels.

The main differences between " normal " collagen or elastic fibres and what we have called " elastotically degenerated collagen " have been detailed fully in other studies (Gillman *et al.*, 1954). In summary these differences are :

(1) The fibres are coarser and wavier than normal collagen bundles and may even be granular and clumped.

(2) They are distinguishable from collagen in that they are :

(*a*) Basophilic, with haematoxylin and other basic dyes.

(*b*) Heavily stained with recognised elastic stains such as Verhoeff's, Taenzer-Unna-Orcein and Weigert's methods.

(*c*) Chromophobic to the anilin blue in Mallory's triple connective-tissue stain, and almost so to the haematoxylin derivatives in Mallory's phosphotungstic acid haematoxylin.

(3) Although they stain with all recognised elastic stains, the elastotically degenerated fibres can be differentiated from " true " elastic fibres or membranes thus :

(*a*) By their morphology, although they approach in thickness normal elastic " membranes ".

(*b*) Whereas they stain with some of the elastic fibre stains, they fail to stain with other dyes which colour thick elastic membranes present in normal blood vessels and in dermis.

(*c*) On the other hand, they also stain with other dyes which do *not* colour " normal " elastic fibres or membranes (Gillman *et al.*, 1954).

It seemed unlikely, from the study of our material, that these long stretches or smaller clumps of elastotically degenerated fibres, encountered in donor sites treated with homo-dermis, could have been derived from the collagen fibres of the grafted homo-dermis itself. It is probable that this possibility can only be excluded by the use of radio-actively labelled homo-dermal grafts. However, we tend to the view that these elastotically degenerated fibres, which appear in healed Thiersch-graft donor sites treated with homo-dermal grafts, result from disturbances in normal collagen formation in the experimental site. We therefore refer to them as " elastotically degenerated collagen ". Proof of this view is, of course, still to be provided.

Our interest in these elastotically degenerated fibres lies in two main points :

Wherever such abnormal fibres were encountered, they were invariably associated with spurs of epithelium growing downwards either directly through, or more commonly around the edges of these fibre masses (Fig. 6 and 7). These latter epidermal spurs could become quite extensive and thick and were often seen to undermine completely the elastotically degenerated fibre masses. The latter thus often came to be completely, or almost completely, surrounded by a ring of epithelium. That portion of the surrounding epidermal ring immediately underlying the abnormal fibres, thickens and then seems to undergo internal keratinisation. Ultimately the elastotically degenerated fibre mass is sloughed off in flakes together with the original surface epithelium and that part of the deep epidermal bar which came to lie between the zone of internal keratinisation and the elastotically degenerated fibres. With recurrent sloughing, in small flakes, of the elastotically degenerated fibres encapsulated in epithelium, the *new* surface epithelium is represented by that portion of the new epidermal bar which lies deep to the zone of internal keratinisation. This process of sloughing of clumps of elastotically degenerated fibres closely resembles the sloughing of a dried blood clot or " scab " which forms over an excised wound or an abrasion (Loeb, 1898 ; Gillman *et al.*, 1953).

### EXPLANATION OF PLATES

FIG. 1.—Section of ten-day-old untreated healing Thiersch-graft donor site showing epidermal hyperplasia and " invasion " of *new* connective tissue by epidermal " pseudo-pegs". Weigert's elastic stain, haematoxylin-eosin. × 32.

FIG. 2.—Section of seventeen-day-old untreated Thiersch-graft donor site to show removal of serrations on lower surface of regenerated epidermis and the formation, in the new sub-epithelial connective tissues, of epithelial pearls and related foreign body reactions. Iron-haematoxylin. × 32.

FIG. 3.—Shows the well-marked epidermal hyperplasia and invasiveness occurring in relation to a small fragment of trypsin-treated homo-dermal graft (H.D.G.) which has become encapsulated in host epithelium at about the ninth post-operative day. Note that the epidermal hyperplasia is not localised only to the area immediately below the graft, but extends for some distance around the graft as partially shown at right of figure. Mallory's triple. × 12.

FIG. 4.—Showing the marked hyperplasia and invasiveness which is regularly encountered, at about the seventeenth post-operative day, in those parts of a Thiersch-graft donor site which had previously been treated with trypsin-treated homo-dermis. The degree of epithelial hyperactivity and invasiveness can be well appreciated by comparison of this figure with Fig. 2. Verhoeff's elastic stain. × 32.

FIG. 5.—Another example of the morphology and extent of the epidermal hyperplasia and invasiveness which supervenes in Thiersch-graft donor sites, seventeen days after the application thereto of trypsin-treated homo-dermal grafts. Such epidermal hyperplasias resemble, temporarily, local benign neoplasias which are subsequently eliminated. Weigert's elastic stain, Haematoxylin-eosin. × 32.

FIG. 6.—An example of localised areas of " elastotic degeneration " of collagen which are regularly encountered, some forty days after the healing of a Thiersch-graft donor site in areas which have been previously treated with trypsin-treated homo-dermis. Note also the tendency of epidermal spurs to invade the dermis at the edges and even through the substance of such elastotically degenerated plaques. Stained as for Fig. 5. × 43.

FIG. 7.—Another area of " elastotically degenerated " collagen appearing in a healed Thiersch-graft donor site some eighty days following the application thereto of trypsin-treated homo-dermis. The persistence of hyperplasia and invasions of the epidermis into such elastotically degenerated plaques and around the edges thereof is clearly evident. Stained as for Fig. 5. × 72.

FIG. 8.—An area of epidermal hyperplasia and invasiveness associated with a known malignant tumour of the skin, for comparison with Fig. 4 and 5 above. The invasive epidermal mass at the left of this figure consists of malignant cells. Haematoxylin-eosin. × 43.

FIG. 9.—Another specimen of known epidermal carcinoma to show the invading epithelium, epithelial plaque-formation, early epithelial nest-formation and the strong round-cell infiltration evoked by this invasive epidermis. The hyperplasia of the epidermis and the formation of pseudo-rete pegs is also well demonstrated at the left of this figure. For comparison with Fig. 4 and 5. Haematoxylin-eosin. × 32.

FIG. 10.—Skin of dorsum of the hand from a case of chronic, pre-malignant ultraviolet irradiation dermatitis ; showing the epidermal hyperplasia, invasion of short epidermal pseudo-pegs, and the associated large mass of elastotically degenerated collagen below. Between this elastotically degenerated collagen bed and the epidermis there has now appeared a zone of round-cell infiltration and new connective-tissue formation. For comparison with Fig. 6 and 7 above. Weigert's elastic stain, haematoxylin-eosin. × 32.

FIG. 11.—Showing similar mass of elastotically degenerated collagen lying in an extensive X-radiation scar. The atrophic nature of the overlying epithelium with the formation of small pseudo-peg invasions of the dermis are also clearly depicted. For comparison with Fig. 6, 7 and 10 above. Mallory's phospotungstic acid haematoxylin. × 32.

FIG. 12.—Showing a large mass of elastotically degenerated collagen in a twenty-seven-year-old burn-scar which has undergone malignant change following recent minor injury thereto. Once more the abnormal overlying epidermis and the formation of numerous new pseudo-pegs of invading epidermis are clearly depicted, together with true neoplasia (at left). Orcein-haematoxylin. × 72.

1.
2.
H.D.G.
3.
4.
5.
6.

Gillman, Penn, Bronks and Roux.

Thus, these elastotically degenerated fibre masses once produced, even in "normal" skin, seem to evoke a definite invasive, albeit localised and benign "new growth" of epidermis which is evanescent but recurrent.

The second reason for interest in these elastotically degenerated collagen masses lies in the fact that we have yet to see a skin cancer in man which is *not* associated with similar elastotically degenerated collagen masses—of greater or lesser extent. Examples of such elastotically degenerated fibre masses, in precancerous and frankly cancerous skin-lesions caused by chronic ultraviolet, X-ray or radium-irradiation, and also in association with a malignant old burn scar, are depicted in Fig. 10, 11 and 12. The morphology of the associated non-malignant and malignant epidermal hyperplasias and invasions of the dermis are portrayed in Fig. 8, 9 and 12, for comparison with the reactions produced experimentally in normal, non-irradiated human skin (Fig. 7 and 8).

The morphological, tinctorial and histochemical reactions of the experimentally induced elastotic degenerations are identical with those occurring in pre-cancerous or cancerous skin lesions in man. We have, however, also encountered similar elastotically degenerated collagen in the so-called elastica mimica of normal adult facial skin, in non-malignant old burn scars, and in degeneratory processes in the connective tissues, at least of arteries, and in the tunica propria of the gall-bladder.

### DISCUSSION.

In the introductory section of this study, we have outlined several problems, relating to the histogenesis of skin cancers, which require solution if progress is to be made in understanding carcinogenesis. The findings which we have here presented permit the following broad general statements :

1. Epidermal invasions of the dermis are normal concomitants of the repair of acute and chronic skin injuries.
2. The supervention of such epidermal invasions seems to be related, directly or indirectly, to the activity of the connective tissue during certain stages of wound healing.
3. The invasive activity of the epidermis, during normal wound healing, seems to be brought under control by the activity of the connective tissue, the regeneration of which appears to be initiated by epithelial regeneration.
4. Disturbances of the connective tissue, in healing wounds, may *alone* bring about markedly increased, albeit an evanescent hyperplasia and invasiveness of the epithelium.
5. Disturbances of connective tissue, at least during the early stages of healing, may result in prolonged alterations in the connective tissue in the healed site, with associated epidermal activity, of major or a minor order, but persisting for at least eighty days.
6. Dermal changes, apparently identical in quality (but differing in extent, duration and no doubt also in mode of origin), are also consistently encountered in many degeneratory changes in the human skin associated with ageing, chronic radiation injuries or with pre-cancerous or frankly cancerous lesions.

In our experiments the collagen placed in the healing donor site (as a trypsin-treated homo-dermal graft) was abnormal in that, firstly, it was a homo-graft and, secondly, it had been previously subjected to digestion with trypsin. We now know that trypsin treatment is not essential and that the dermal fragments (from

*untreated* split-skin homografts) which remain after epidermal sloughing (Medawar, 1945) may, in time, evoke similar reactions, but to a lesser degree. In ageing skin, as in skin damaged acutely or chronically by various irradiations or by burning, similar dermal changes result from local or systemic alterations in the metabolism of the connective tissues in general, and of collagen in the repaired site in particular. Also, in the latter circumstances, elastotic degeneration of collagen is much more extensive and prolonged than in the conditions of our experiments reported above.

Whether *repeated* applications of abnormal connective tissue to traumatised non-irradiated young skin will ultimately permit the experimental production of epidermal neoplasms in animals or in man is still under investigation in this laboratory. We can state, at this stage, that composite healthy auto-grafts, and auto- or homo-grafts of epidermal or dermal tissue do profoundly alter the healing of standard experimentally inflicted excised wounds in rabbits. Either the epidermal or the dermal components may be significantly affected during healing by the application of various types of auto- and homografts. We are led to conclude, therefore, that the reactions of the epidermis in healing and probably also in carcinogenesis cannot be studied in isolation from the related connective tissue changes, since changes in the latter may profoundly influence the epidermis.

As for the second problem raised in the introduction, *i.e.* whether or not skin carcinoma invariably arises from preformed hair follicles, we feel that our studies of healing wounds and of carcinogenesis in man and in animals allow us to make certain suggestions.

Wolbach (1951) stated that neogenesis of hair follicles does not occur in the mouse, and, on the other hand, that neoplasms develop from : " . . . hair follicles which have lost papillae rest cells . . . the follicle deprived of its papilla is no longer the source of new follicle formations . . . the explanation of focal origin of papillomas awaits better understanding of the conditions which result in the destruction of papilla cells."

To revert to our previously recorded findings on healing wounds—as stated above, epidermal " pseudo-pegs ", invading the connective tissue, consistently occur during wound healing in man and in more hairy animals, such as mice and rabbits. That neogenesis, at least of sebaceous glands and hair follicles, can occur in healed wounds or in scar epithelium now seems to be clearly established (*vide supra*). It may well be that the formation of invasive epithelial pseudo-pegs represents a reversion to an earlier ontogenetic behaviour characterising developing skin in the embryo (in man) or early post-natally in rodents.

Our findings in methylcholanthrene-treated skin of mice lead us to believe that the *original* hair follicles and sebaceous glands are usually *completely* destroyed by chronic treatment with this carcinogen. It seems that if new hair follicles and sebaceous glands form in sites severely injured by the carcinogen that they do so *de novo* from the equivalents of those invasive epidermal pseudo-pegs which we have described above, as occurring invariably during normal wound healing.

The reformation of hair demands at least two essentials :

(1) The presence of connective tissue competent to respond to epidermal down-growths, by forming new hair papillae. Even during embryological development both epidermal and dermal components are indispensable for normal hair formation.

(2) An epidermal invasion of the dermis capable, firstly, of acting as an evocator of connective tissue to form a hair papilla and, secondly, of responding to the organizing actions of such dermal papillae.

There is ample evidence in the literature (Orr, 1939 ; Marchant and Orr, 1953 ; Ma, 1949) and from our own experimental studies that the dermis is profoundly altered by the application of carcinogens, and that these changes in the connective tissues precede epidermal neoplasia. It seems possible, therefore, that such dermal damage may result in an inability on behalf of the connective tissues to respond to the normally healing epidermal down-growths with subsequent failure to reform hair papillae. The latter seem to play an important rôle in organising the regenerated epidermal " Anlagen " into new hairs. Under these circumstances epidermal invasions occurring normally during healing would not be adequately controlled and may then, perhaps, become neoplastic.

In view of what we have said above, we submit, as a possibility, that carcinomas in man and in animals may result in any of those circumstances in which there are attempts at epidermal regeneration, with the associated normal invasions of the dermis by epidermal pseudo-pegs (or pseudo-epidermal hair " Anlagen " in animals), which *fail* to evoke in the dermis either (*a*) fibrosis with subsequent elimination of the pseudo-pegs, as in normal wound healing in man, or (*b*) hair-follicle formation with consequent organisation of the epidermal " invasions " into normal *new* hair follicles and/or sebaceous glands.

Most of the investigators who have considered alterations in the dermis as playing a rôle in the pathogenesis of skin carcinomas have usually stated that these dermal changes operate by interfering progressively with the nutrition of the epidermis (Wolbach, 1909 ; Vernoni, 1951). We have shown that alterations in the dermis may *alone* evoke marked hyperplasia and invasiveness of the epidermis. On the basis of these findings in man, as well as from our experimental work, we have suggested here a totally different manner by which damage to the *dermis* may contribute towards the histogenesis of skin cancers, namely, by failing firstly (in rodents) to form hair papillae and as a result to organise epidermal pseudo-hair " Anlagen " into hairs, or secondly (in man) to induce new sebaceous gland formation or to eliminate the epidermal pseudo-pegs which normally develop during healing.

That the connective tissues may play an important rôle in determining the fate of epidermal invasion of the dermis is shown by several experimental studies. In his classic studies of the reactions of auto- and homo-grafts in rabbits Medawar (1944) stated (p. 181) that in autografts : " Not merely the slope, but also the pattern of the mature hair follicles is controlled by the structure of the collagenous endoskeleton of the original graft. If the graft contained multiple nests of hair follicles, multiple nests are developed when the hairs are newly formed."

In the scar-like tissue surrounding auto-grafts, and over which epithelium generated from these grafts spreads, Medawar found that new hairs were *not* formed, and he states (p. 181) : " . . . this is because the connective tissue endoskeleton is not of the right pattern, and not because the new epithelium, however ' immature ' it may appear to be, lacks any part of its normal capacity to manufacture new hairs."

Subsequently, in the same study, Medawar recorded the fate of the dermal pad, remaining after the epidermis of a homo-graft had sloughed. He stated (p. 186) : " Almost as soon as native epithelium reaches the foreign dermis, whether it approaches it from above or from below, hair-follicle primordia are laid down. Evidently skin epithelium, however 'immature', has the power to manufacture hairs provided that it reaches a depot of collagen fibres of the right size and pattern of packing."

The possibility also exists that the failure of dermal tissue to promote new hair follicle formation may be due not only to unfavourable structural organisation of these connective tissues (as suggested by Medawar, 1944) but also to some *chemical* differences. More recently, Cairns and Saunders (1954) have shown that ectoderm-free *leg* mesoderm, implanted into the *wings* of chick embryos, influence the determination of the specific morphogenetic activities of the overlying ectodermal cells of the wing into scales, claws and feathers, characteristic of the leg. Teir, Voutilainen and Kiljunen (1954), on the other hand, claim to have induced neoplasia with methylcholanthrene in the normally resistant rat by simultaneously injecting embryonic skin suspensions. These studies provide indications of the importance of mesodermal derivatives in determining the direction of differentiation of foetal and even of adult surface ectoderm.

Both (or either) the state of the epidermis or of the underlying dermis may apparently be responsible for repeated epidermal pseudo-peg formation (*vide supra*), neogenesis of hair follicles and/or sebaceous glands or, finally, epidermal neoplasia. Viewed in the light of the above discussion, it would seem that the dermis plays at least a most important if not a dominant rôle in determining whether or not carcinoma will supervene in epidermis previously activated to regeneration in response to the effects of physical and/or chemical trauma.

### SUMMARY.

1. During normal healing of Thiersch-graft donor sites in man epithelial "invasions" of the underlying connective tissues occur consistently. Such normal epidermal invasions can be markedly stimulated so that by the seventeenth post-operative day the epithelium, in healing donor sites, may for a while resemble a localised epidermal new growth. Such epidermal hyperplasia is followed, some weeks later, by abnormalities in the connective tissue in the healed donor site.

2. The epidermal hyperplasia, and the subsequent alteration in the related connective tissues mentioned above, can regularly be produced in human subjects by the application to fresh Thiersch-graft donor sites of trypsin-treated homo-dermal grafts. It is suggested that these changes are attributable to the trypsin-treated homo-grafts acting as "foreign" or abnormal collagen.

3. Alterations in the *connective tissues* of the healed donor sites were also found to occur some weeks after the initial operation. These changes are shown to be due to the development of abnormal fibres which stain with all known elastic tissue stains but which, on the basis of various criteria, are nevertheless considered to be "elastotically degenerated" collagen. These elastotically degenerated fibres are shown to be consistently related to intermittent, repeated and localised epidermal invasions of the dermis.

4. It is shown that similar elastotic degeneration of collagen is invariably present in the dermis in many degenerative skin conditions (due to chronic irradiations) which may and frequently do become pre-cancerous or frankly cancerous.

5. It is suggested that the elastotic degeneration of dermal collagen may play an important rôle in the pathogenesis of skin cancers. A possible explanation for the development of carcinomas in chronically traumatised skin is suggested, which maintains that carcinomas supervene when fibrosis or degeneration prevents the dermal tissues either from eliminating or, alternately, from inducing hair formation in those epidermal pseudo-pegs which invade the dermis during normal repair. This suggestion allows for some understanding and further experimental testing of the view that epidermal carcinogenesis is directly related to hair regeneration in man and in animals.

We are glad to acknowledge the very generous grant from the Schlesinger Organisation, which made the execution and the presentation of this work possible. We also wish to express our thanks to Mrs. Florence Powell for her grant in aid of a Cancer Research Library. Mrs. B. H. Robinow's assistance in obtaining journals, under very difficult circumstances, was invaluable and is gratefully acknowledged. To Miss Patricia R. Low, Miss Phyllis Bilbrough and Mrs. Adele Hart we owe our sincerest thanks for their expert and willing technical assistance. Mr. R. Stuart prepared the prints of the photomicrographs. We also wish to record our appreciation of Mrs. Herzog's patient assistance throughout this investigation.

### REFERENCES.

Andreasen, E.—(1953) *Acta path. microbiol. scand.*, **32**, fasc. 1, 157.

*Idem* and Engelbreth-Holm, J.—(1953) *Ibid.*, **32**, fasc. 1, 165.

Billingham, R. E. and Reynolds, J.—(1952) *Brit. J. plast. Surg.*, **5**, 25.

Breedis, C.—(1954) *Cancer Res.*, **14**, 575.

Brown, J. B., McDowell, F. and Fryer, M. P.—(1949) *Surg. Gynec. Obstet.*, **88**, 609.

Cairns, J. M. and Saunders, W. Jr.—(1954) *J. exp. Zool.*, **127**, No. 2.

Chase, H. B.—(1954) *Physiol. Rev.*, **34**, 113.

*Idem* and Montagna, W.—(1951) *Proc. Soc. exp. Biol.*, *N.Y.*, **76**, 35.

Foot, N. C.—(1947) *Amer. J. Path.*, **23**, 1.—(1951) *Ann. N.Y. Acad. Sci.*, **53**, 749.

Gillman, J. and Gillman, T.—(1951) 'Perspectives in Human Malnutrition.' New York (Grune & Stratton).

Gillman, T., Penn, J., Bronks, D. and Roux, M.—(1953) *Brit. J. plast. Surg.*, **6**, 153. — (1954) *Nature*, **174**, 789.—(1955) *Brit. J. Surg.* (in press).

Hall, I. W. and Herxheimer, G.—(1905) ' Methods of Morbid Histology and Clinical Pathology.' (Green & Sons.)

Lever, W. F.—(1948*a*) *Arch. Derm. Syph. N.Y.*, **57**, 679.—(1948*b*) *Ibid.*, **57**, 709.

Lillie, R. D.—(1952) ' Histopathologic Technic and Practical Biochemistry.' New York (Blakiston Comp.).

Loeb, L.—(1898) *Arch. EntwMech. Org.*, **6**, 297.

Ma, C. K.—(1949) *Cancer Res.*, **9**, 481.

Mallory, F. B.—(1914) ' Principles of Pathologic Histology,' 371. Philadelphia (W. B. Saunders).

Marchant, J. and Orr, J. W.—(1953) *Brit. J. Cancer*, **7**, 329.

Medawar, P. B.—(1944) *J. Anat. Lond.*, **78**, 176.—(1945) *Ibid.*, **79**, 157.

Montgomery, H.—(1946) ' Pathologic Histology of Radiodermatitis,' Chapter 23, in ' X-Rays and Radium in Treatment of Disease of the Skin,' 4th ed. Philadelphia (Lea & Febiger).

Orr, J. M.—(1939) *J. Path. Bact.*, **49**, 157.

Puhr, L.—(1935) Quoted by W. N. Goldsmith in ' Recent Advances in Dermatology ' (1936). London (J. & A. Churchill Ltd.).

Rinehart, J. F. and Abul-Haj, S. K.—(1951) *Arch. Path.*, **52**, 189.

Teir, H., Voutilainen, A. and Kiljunen, A.—(1954) *Acta path. microbiol. scand.*, **34**, fasc. 3, 218.

Teloh, H. A., Mason, M. L. and Wheelock, M. C.—(1950) *Surg. Gynec. Obstet.*, **90**, 335.

Vernoni, G.—(1951) *Sci. med.*, *Ital.*, **2**, 373.

Wolbach, S. B.—(1909) *J. med. Res.*, **21**, 415.—(1951) *Ann. N.Y. Acad. Sci.*, **53**, 517.